# Supplementary material for: Functional analysis of genetic variants in the high-risk breast cancer susceptibility gene PALB2
Source: Nat Commun. 2019 Nov 22;10:5296. doi: 10.1038/s41467-019-13194-2 (PMC6876638; doi:10.1038/s41467-019-13194-2)
Supplement: Supplementary file 1 — Supplementary Information [file 41467_2019_13194_MOESM1_ESM.pdf]

Supplementary information

Functional analysis of genetic variants in the high-risk breast cancer susceptibility gene *PALB2*

Rick A.C.M. Boonen, Amélie Rodrigue, Chantal Stoepker, Wouter W. Wiegant, Bas Vroling, Milan Sharma, Magdalena B. Rother, Nandi Celosse, Maaike P.G. Vreeswijk, Fergus Couch, Jacques Simard, Peter Devilee, Jean-Yves Masson and Haico van Attikum*

* Corresponding author. E-mail: h.van.attikum@lumc.nl

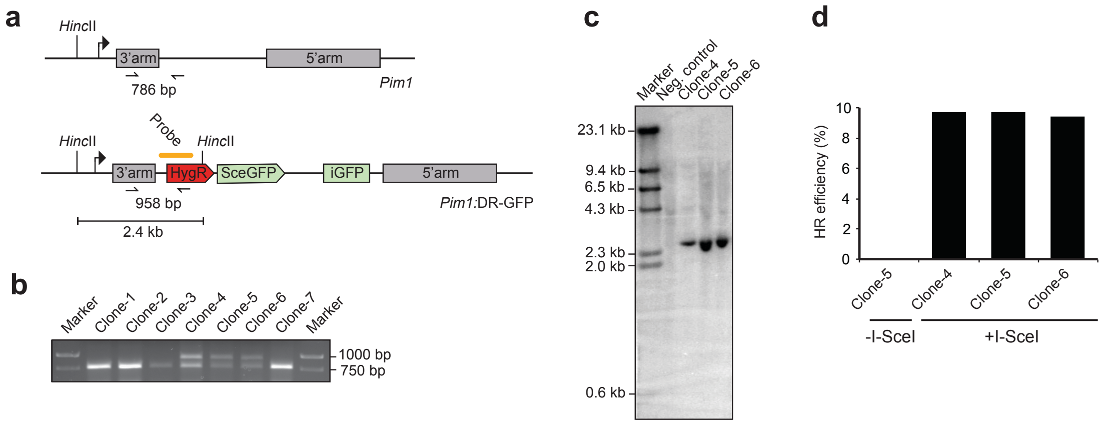

Supplementary Fig. 1 Stable integration of the DR-GFP reporter at the *Pim1* locus in mES cells. **a** Schematic showing the *Pim1* locus (upper) and *Pim1* locus with an integrated DR-GFP reporter (*Pim1:*DR-GFP; lower) in mES cells. Integration is directed by the 3' and 5' homology arms. Correct integration of the reporter results in expression of a hygromycin resistance marker under control of the endogenous *Pim1* promoter (not shown). Correct integration was examined by PCR and Southern blot analysis using the indicated primers, as well as a probe and restriction enzymes, respectively. **b** PCR analysis of genomic DNA from hygromycin-resistant mES cell clones obtained after targeting the *Pim1* locus with a DR-GFP cassette using primers indicated in (**a**). Clone 4-6 show correct integration of DR-GFP at a *Pim1* allele (as evidenced by the appearance of a 958 bp band). **c** Southern blot analysis of *Hinc*II-digested genomic DNA from mES cell clones 4-6 from (**b**) using the probe shown in A. Single copy genomic integration at a *Pim1* allele is observed in all three clones (as evidenced by the appearance of a 2.4 kb band). **d** DR-GFP assay DR-GFP assay in clone 4-6 from (**b**) and (**c**). Cells were transfected with an I-SceI and mCherry co-expression vector, or with an mCherry expression vector only. GFP expression was monitored by FACS. Data represent the absolute percentage of GFP-positive cells among the mCherry-positive-cells. Source data are provided as a Source Data file.

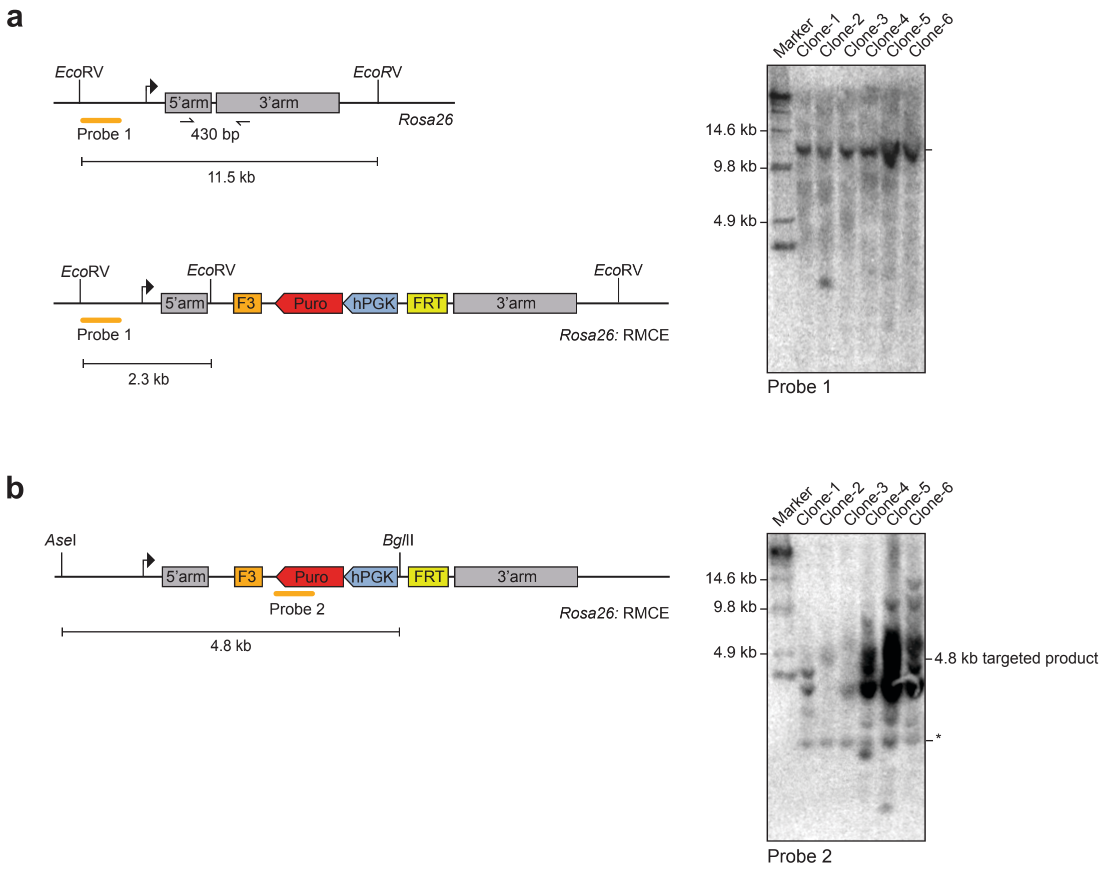

Supplementary Fig. 2 Stable integration of the RMCE acceptor cassette at the *Rosa26* locus in mES cells carrying DR-GFP. **a** Schematic showing the *Rosa26* locus (upper left) and *Rosa26* locus with an integrated RMCE acceptor cassette (*Rosa26:*RMCE; lower left) in mES cells. Integration is directed by the 3' and 5' homology arms. Correct integration of the RMCE acceptor cassette results in expression of a puromycin resistance marker under control of the PGK1 promoter. Correct integration was examined by Southern blot analysis of *Eco*RV-digested genomic DNA from mES cell clones 1-6 using the indicated probe (right). Single copy genomic integration at a *Rosa26* allele is observed in clone 2 (as evidenced by the appearance of a 2.3 kb band). **b** Schematic as in (**a**), except that a different probe and different restriction sites for Southern blot analysis are shown (left). Correct integration was examined by Southern blot analysis of *Ase*I- and *Bgl*II-digested genomic DNA from mES cell clones 1-6 using the indicated probe (right). Single copy genomic integration at a *Rosa26* allele is observed in clone 2 (as evidenced by the appearance of a 4.8 kb band).

a Sequence analysis *Trp53*^{KO}

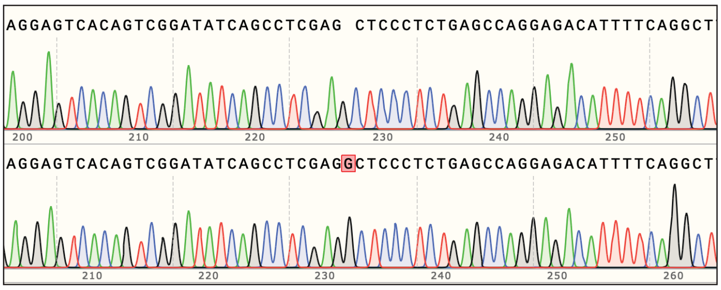

b TIDE analysis *Trp53*^{KO}

Total efficiency = 95.0 %

R^2 = 0.95

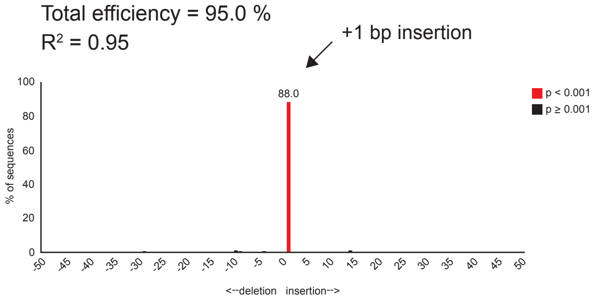

c Sequence analysis *Palb2*^{KO}

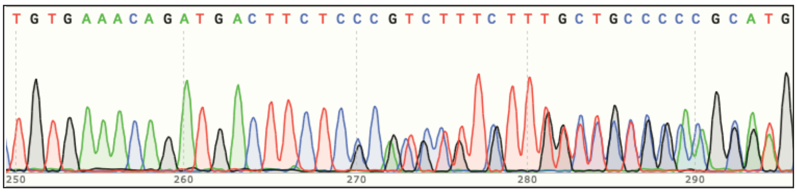

d TIDE analysis *Palb2*^{KO}

Total efficiency = 87.0 %

R^2 = 0.87

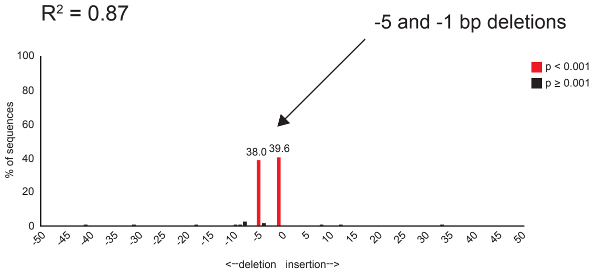

Supplementary Fig. 3 Validation of *Trp53*^{KO}/*Palb2*^{KO} mES cells. **A**, Sequence alignment of a fragment of exon 1 of the *Trp53* gene showing a +1 bp (guanine) insertion in *Trp53*^{KO} clone 3. **b** TIDE analysis confirming the +1 bp insertion in exon 1 of the *Trp53* gene in *Trp53*^{KO} clone 3. **c** Sequence alignment of a fragment of exon 4 of the *Palb2* gene showing -5 bp and -1 bp deletions in the *Palb2*^{KO} clone. **d** TIDE analysis confirming -1 and -5 bp deletions in exon 4 of the *Palb2* gene in the *Palb2*^{KO} clone.

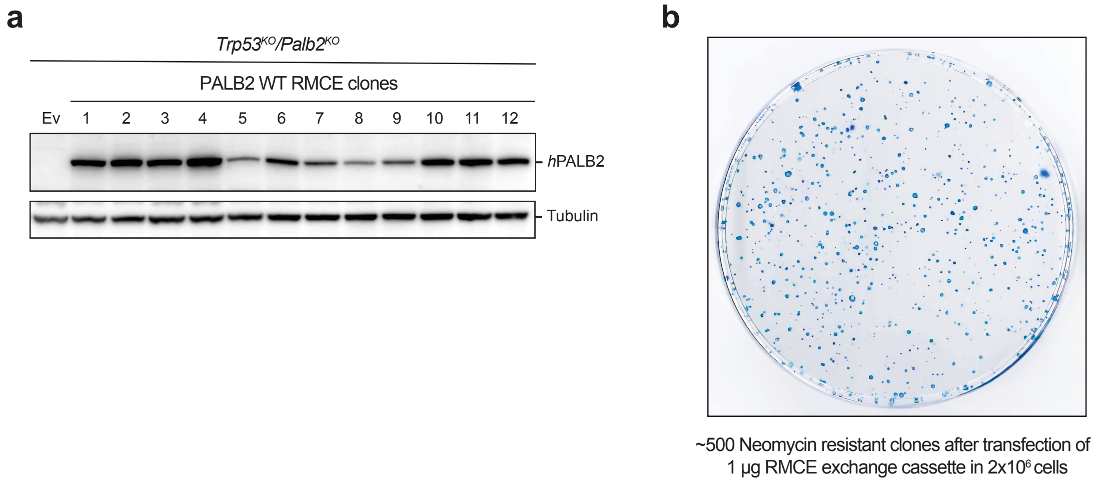

Supplementary Fig. 4 RMCE efficiency in mES cells. **a** Western blot analysis of the expression of wild-type (WT) human *PALB2* in 12 individual *Trp53^{KO}/Palb2^{KO}* mES cell clones using an antibody directed against the N-terminus of PALB2. An empty vector (Ev) served as negative control. Tubulin was a loading control. **b** Representative image of a culture dish with methylene-stained neomycin resistant clones after transfection of *Trp53^{KO}/Palb2^{KO}* mES cells using RMCE exchange cassette. Source data are provided as a Source Data file.

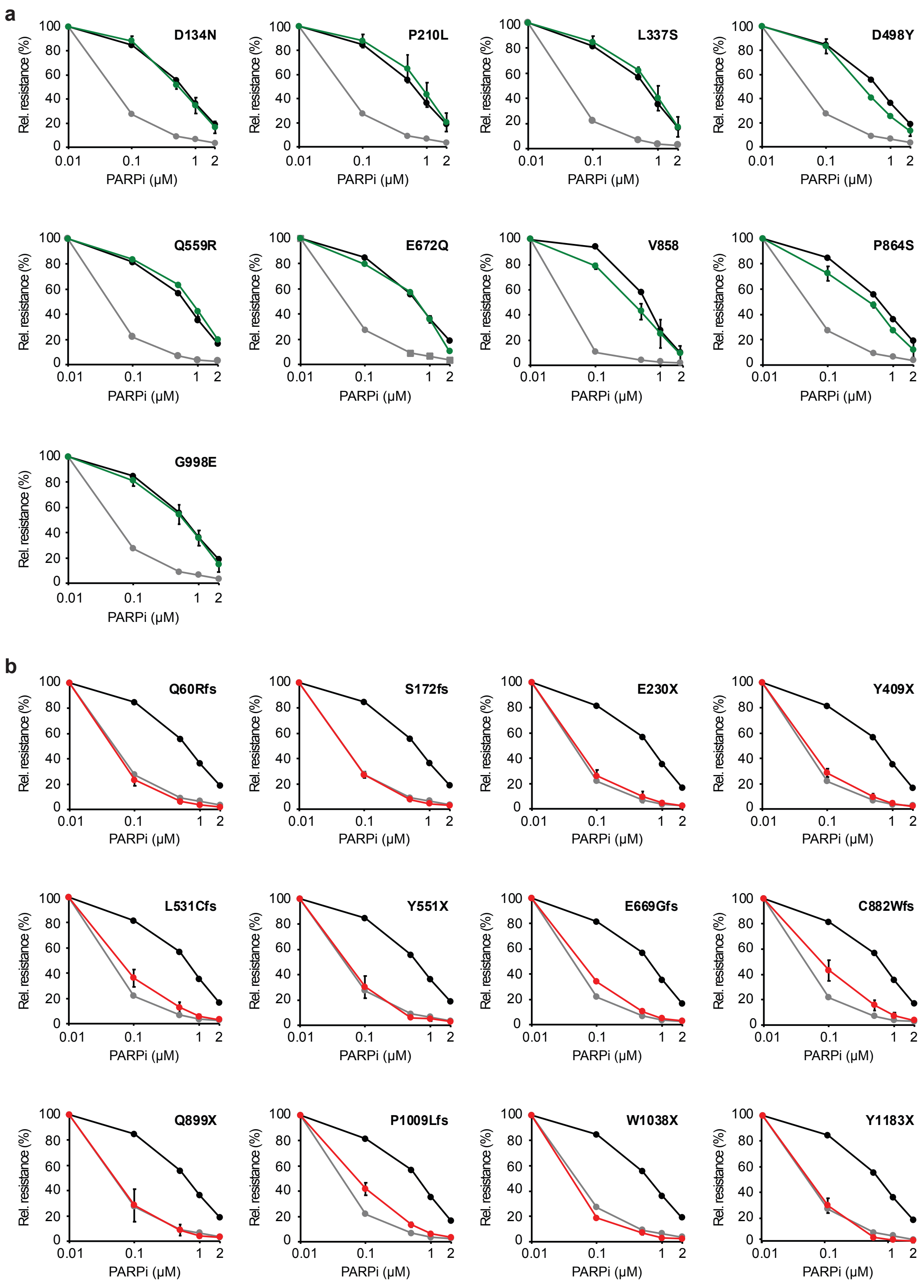

Supplementary Fig. 5 Functional analysis of benign and truncating variants in human *PALB2* by PARPi sensitivity assays. **a** PARP inhibitor (PARPi) sensitivity assay using *Trp53*^{KO}/*Palb2*^{KO} mES cells expressing human *PALB2* variants (or an empty vector control, Ev). Cells were exposed to the indicated concentrations of PARPi for two days.

Cell viability was measured 1 day later using FACS. Data represent the mean percentage of viability/resistance relative to untreated cells (± SEM) from 2 independent experiments. An additional third replicate experiment was performed for PALB2 variant p.P210L. Variants/conditions are categorized by color as either wild type (WT, black), likely benign SNV (green), or empty vector (Ev, grey). Data from the 0.5 µM PARPi concentration are shown in Fig. 3a. **b** as in (**a**), except for truncating variants (red). Source data are provided as a Source Data file.

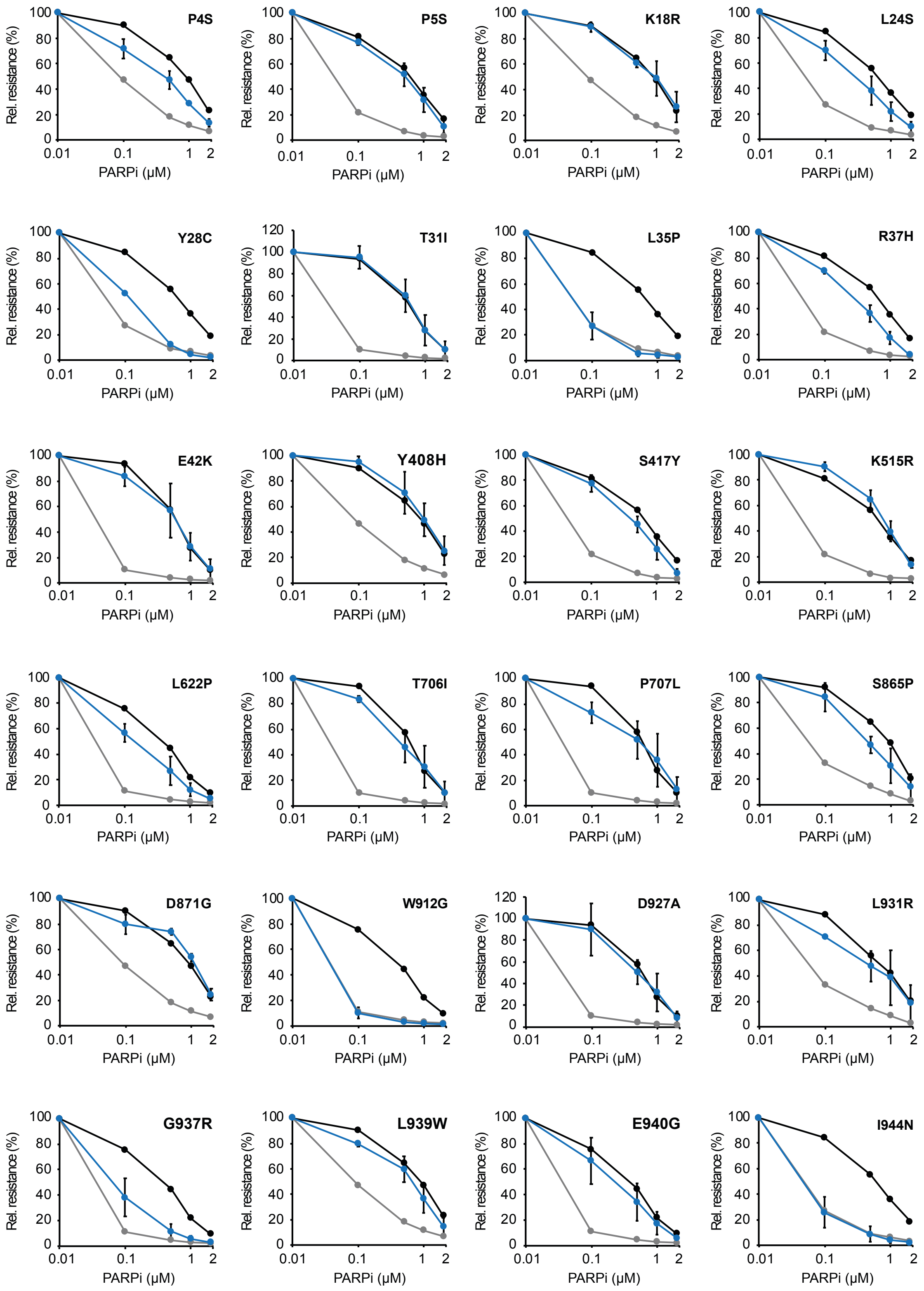

Supplementary Fig. 6 Functional analysis of selected VUS in human *PALB2* by PARPi sensitivity assays. PARP inhibitor (PARPi) sensitivity assay using *Trp53*^{KO}/*Palb2*^{KO} mES cells expressing human *PALB2* variants (or an empty vector control, Ev). Cells were exposed to the indicated concentrations of PARPi for two days.

Cell viability was measured 1 day later using FACS. Data represent the mean percentage of viability/resistance relative to untreated cells (± SEM) from 2 independent experiments. An additional third replicate experiment was performed for PALB2 variants p.P4S and p.L939W and four replicate experiments were performed for p.L24S. Variants/conditions are categorized by color as either wild type (WT, black), VUS (blue), or Ev (grey). Data from the 0.5 µM PARPi concentration are shown in Fig. 3a. Source data are provided as a Source Data file.

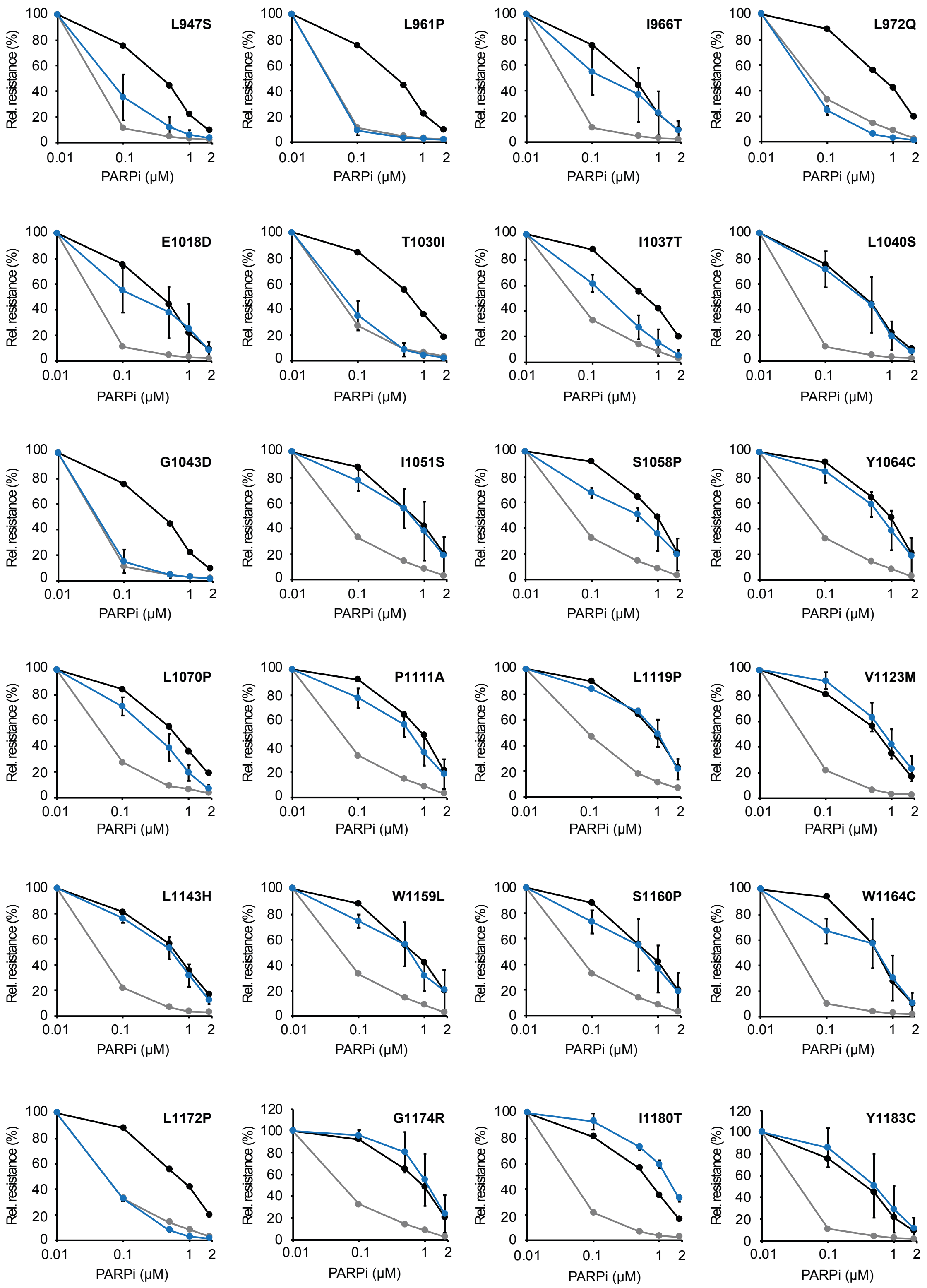

Supplementary Fig. 7 Functional analysis of selected VUS in human *PALB2* by PARPi sensitivity assays. PARPi sensitivity assay using *Trp53*^{KO}/*Palb2*^{KO} mES cells expressing human *PALB2* variants (or an empty vector control, Ev). Cells were exposed to the indicated concentrations of PARPi for two days. Cell viability was

measured 1 day later using FACS. Data represent the mean percentage of viability/resistance relative to untreated cells (± SEM) from 2 independent experiments. An additional third replicate experiment was performed for PALB2 variant p.V1123M, and four replicate experiments were performed for p.L1070P. Variants/conditions are categorized by color as either wild type (WT, black), VUS (blue), or Ev (grey). Data from the 0.5 µM PARPi concentration are shown in Fig. 3a. Source data are provided as a Source Data file.

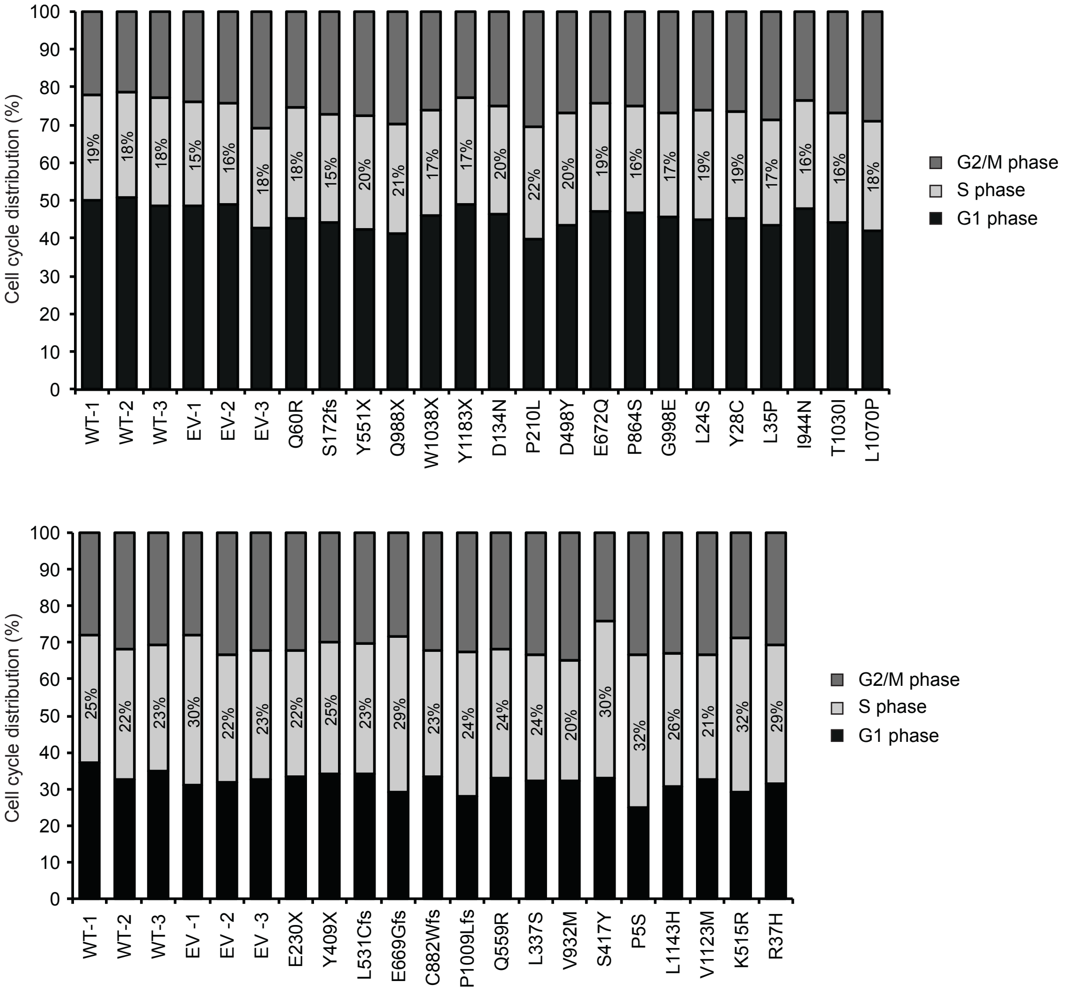

Supplementary Fig. 8 Cell cycle profiles of *Trp53*^{KO}/*Palb2*^{KO} mES cells expressing human *PALB2* variants. Cell cycle profiles are from cells in Fig. 2b. Cells were treated with propidium staining (PI) and analyzed by FACS. Data represent the mean percentage of cell cycle phase distributions from 2 independent measurements. Source data are provided as a Source Data file.

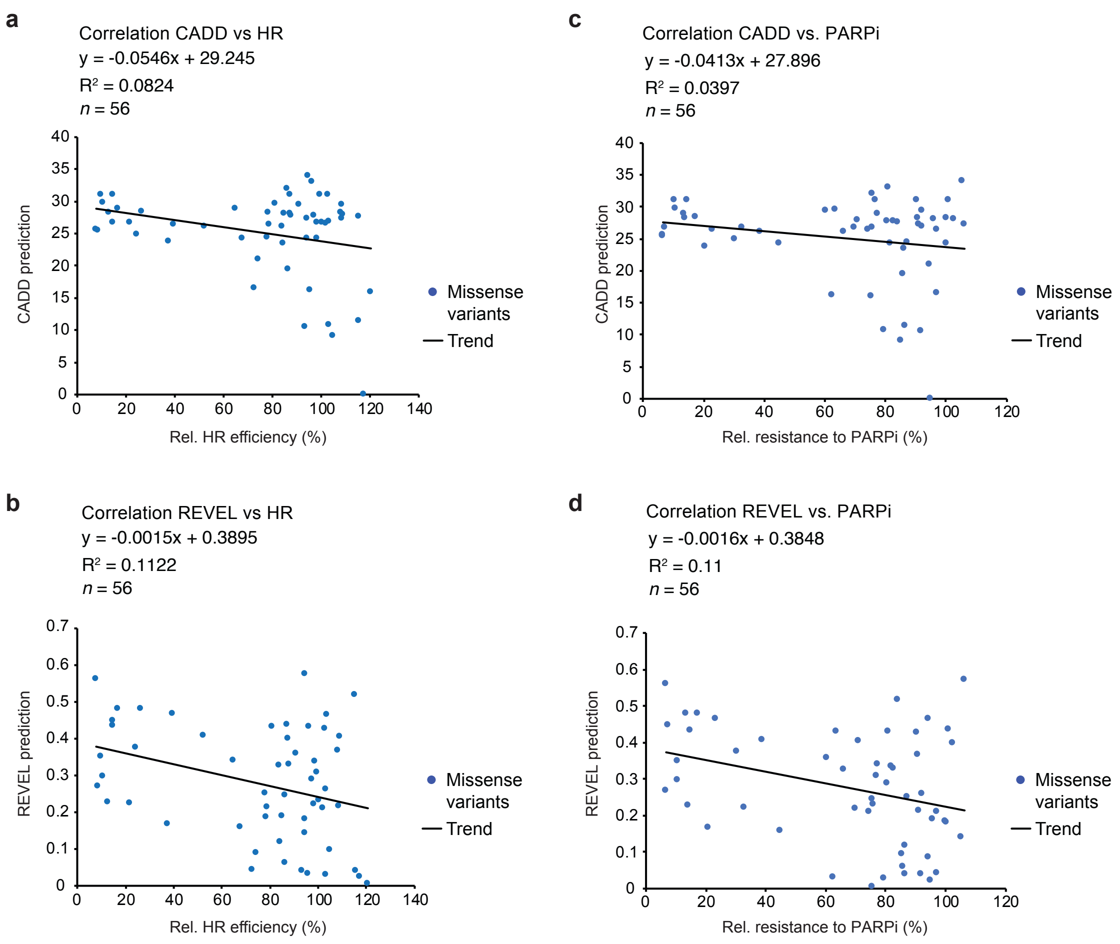

Supplementary Fig. 9 Correlation between *in silico* predictions and the outcome of functional assays for missense variants in human *PALB2*. **a** Scatter plot showing correlation between the *in silico* prediction from CADD and results from the DR-GFP assay in Fig. 2b. **b** Scatter plot showing correlation between the *in silico* prediction from REVEL and results from the DR-GFP assay in Fig. 2b. **c** Scatter plot showing correlation between the *in silico* prediction from CADD and results from the PARPi sensitivity assay in Fig. 3a. **d** Scatter plot showing correlation between the *in silico* prediction from REVEL and results from the PARPi sensitivity assay in Fig. 3a.

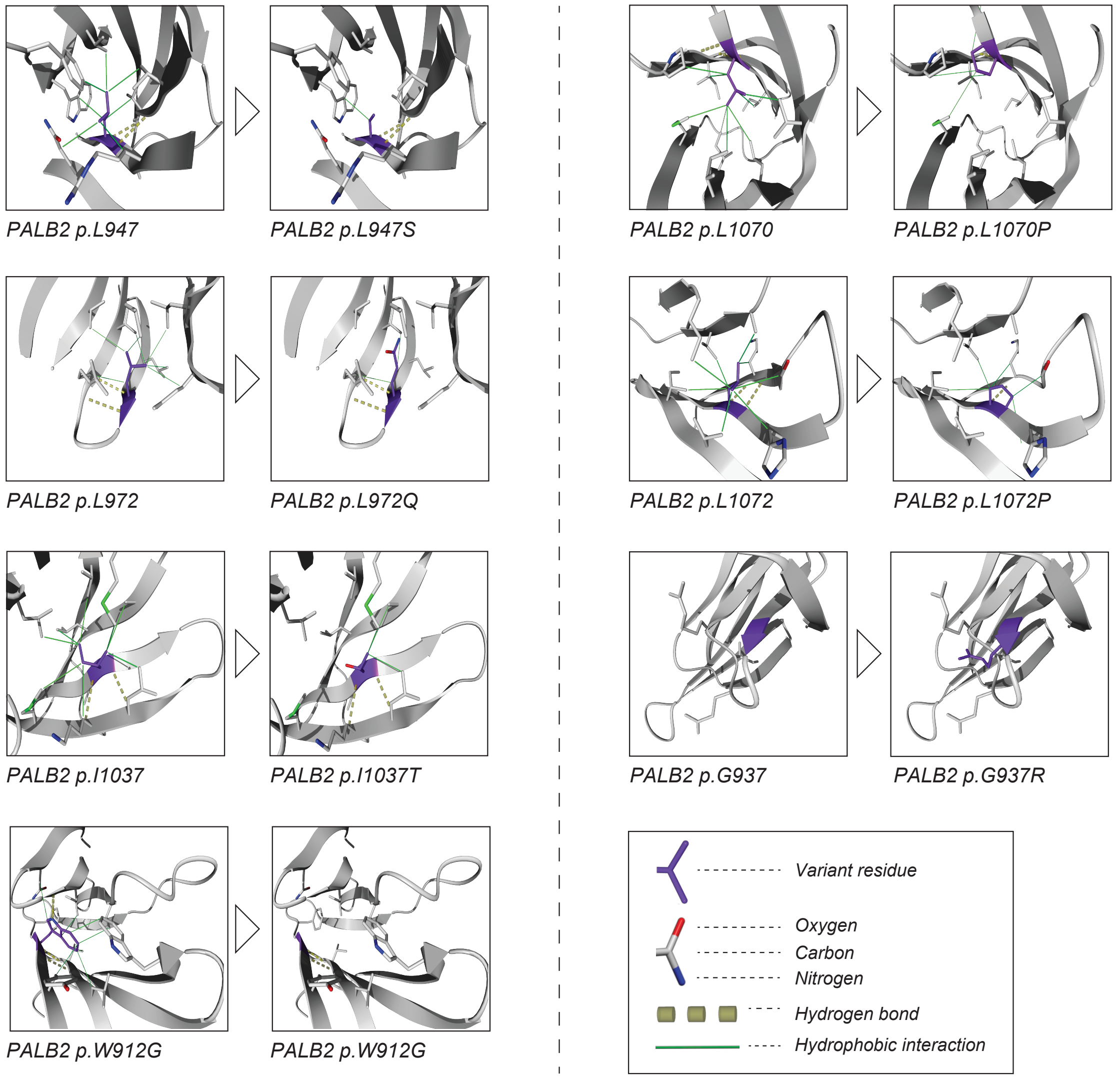

Supplementary Fig. 10 Effect of *PALB2* variants on protein stability. Partial structures of the PALB2 WD40 domain showing the effect of 7 PALB2 variants exhibiting low protein expression as shown in Fig. 4a. Partial structures without and with variant are shown side by side for each variant, indicating loss of stabilizing interactions (but not any possible conformational changes).

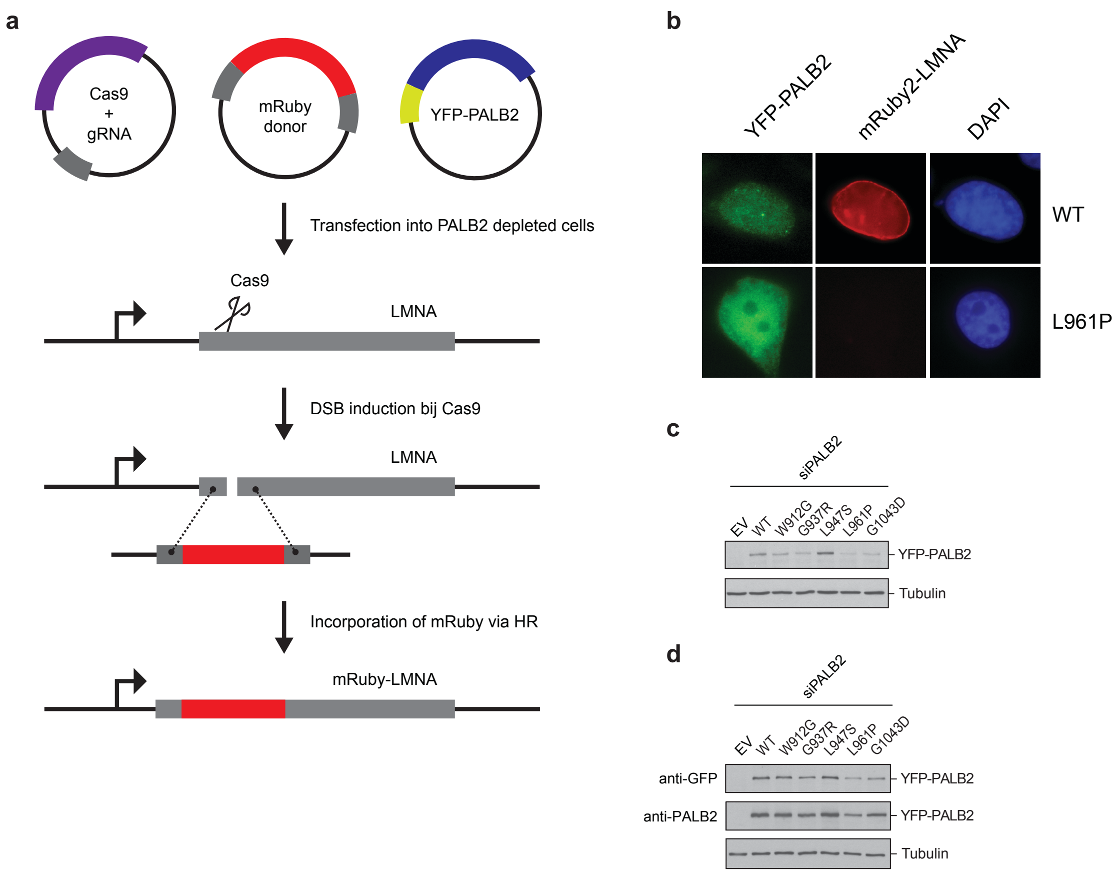

Supplementary Fig. 11 Functional analysis of damaging *PALB2* variants in human cells. **a** Schematic of the CRISPR-LMNA HDR assay in human cells. Homology-directed repair of the Cas9-induced DSB will result in the in-frame integration of mRuby in the first exon of *LMNA*, leading to expression of red fluorescent mRuby-LMNA. The number of mRuby-positive cells is a measure of the HR efficiency. **b** Representative fluorescence microscopy images of mRuby2-LMNA expression after successful homology directed repair (HDR) in a PALB2-depleted U2OS cell complemented with YFP-PALB2-WT (upper), and a cell negative for mRuby2-LMNA expression after complementation with the damaging YFP-PALB2-L961P variant (lower). **c** Western blot analysis of the expression of human *PALB2* variants in si*PALB2*-treated U2OS cells 24 hours after complementation with the indicated siRNA-resistant YFP-*PALB2* variant cDNA constructs. Tubulin was a loading control. **d** Western blot analysis of the expression of human PALB2 variants in si*PALB2*-treated HeLa cells 24 hours after complementation with the indicated siRNA-resistant YFP-PALB2 variant cDNA constructs. Tubulin was a loading control. Source data are provided as a Source Data file.
