# Supplementary material for: Functional analysis of genetic variants in the high-risk breast cancer susceptibility gene PALB2
Source: Nat Commun. 2019 Nov 22;10:5296. doi: 10.1038/s41467-019-13194-2 (PMC6876638; doi:10.1038/s41467-019-13194-2)
Supplement: Supplementary file 3 — Description of Additional Supplementary Files [file 41467_2019_13194_MOESM3_ESM.pdf]

Description of Additional Supplementary Files

File Name: Supplementary Data 1

Description: This Supplementary Data file lists all human PALB2 variants that have been analyzed in this study, as well as the results from *in silico* predictions, DR-GFP, PARPi sensitivity, cisplatin sensitivity and G2/M checkpoint assays.
