# Supplementary material for: Functional analysis of genetic variants in the high-risk breast cancer susceptibility gene PALB2
Source: Nat Commun. 2019 Nov 22;10:5296. doi: 10.1038/s41467-019-13194-2 (PMC6876638; doi:10.1038/s41467-019-13194-2)
Supplement: Supplementary file 5 — Reporting Summary [file 41467_2019_13194_MOESM5_ESM.pdf]

nature research

Corresponding author(s): *Double-blind peer review submissions: write DBPR and your manuscript number here instead of author names.*

Last updated by author(s): *YYYY-MM-DD*

Reporting Summary

Nature Research wishes to improve the reproducibility of the work that we publish. This form provides structure for consistency and transparency in reporting. For further information on Nature Research policies, see Authors & Referees and the Editorial Policy Checklist.

Statistics

For all statistical analyses, confirm that the following items are present in the figure legend, table legend, main text, or Methods section.

n/a	Confirmed	
☐	☒	The exact sample size (*n*) for each experimental group/condition, given as a discrete number and unit of measurement
☐	☒	A statement on whether measurements were taken from distinct samples or whether the same sample was measured repeatedly
☒	☐	The statistical test(s) used AND whether they are one- or two-sided<br>*Only common tests should be described solely by name; describe more complex techniques in the Methods section.*
☐	☒	A description of all covariates tested
☒	☐	A description of any assumptions or corrections, such as tests of normality and adjustment for multiple comparisons
☐	☒	A full description of the statistical parameters including central tendency (e.g. means) or other basic estimates (e.g. regression coefficient) AND variation (e.g. standard deviation) or associated estimates of uncertainty (e.g. confidence intervals)
☒	☐	For null hypothesis testing, the test statistic (e.g. *F*, *t*, *r*) with confidence intervals, effect sizes, degrees of freedom and *P* value noted<br>*Give P values as exact values whenever suitable.*
☒	☐	For Bayesian analysis, information on the choice of priors and Markov chain Monte Carlo settings
☒	☐	For hierarchical and complex designs, identification of the appropriate level for tests and full reporting of outcomes
☒	☐	Estimates of effect sizes (e.g. Cohen's *d*, Pearson's *r*), indicating how they were calculated

Our web collection on statistics for biologists contains articles on many of the points above.

Software and code

Policy information about availability of computer code

Data collection

1. TIDE: Easy quantitative assessment of genome editing by sequence trace decomposition
2. Align GVGD
3. Sorting Intolerant From Tolerant (SIFT)
4. Combined Annotation Dependent Depletion (CADD)
5. Rare Exome Variant Ensemble Learner (REVEL)
6. Polymorphism Phenotyping (PolyPhen)

Data analysis

1. Bio-Rad CFX thermal cycler software (mRNA analysis)
2. Gen5 Data Analysis Software v3.03, BioTek Instruments (PARPi sensitivity in human cells)
3. ZEN 2012 microscopy software (Karyotyping mES cells)
4. Volocity software v6.0.1, Perkin-Elmer Improvision (counting RAD51 foci and assessing intensity)
5. NovoExpress 1.2.5. software (DR-GFP and PARPi assays in mES cells)

For manuscripts utilizing custom algorithms or software that are central to the research but not yet described in published literature, software must be made available to editors/reviewers. We strongly encourage code deposition in a community repository (e.g. GitHub). See the Nature Research guidelines for submitting code & software for further information.

Data

Policy information about availability of data

All manuscripts must include a data availability statement. This statement should provide the following information, where applicable:

- Accession codes, unique identifiers, or web links for publicly available datasets
- A list of figures that have associated raw data
- A description of any restrictions on data availability

n/a

Field-specific reporting

Please select the one below that is the best fit for your research. If you are not sure, read the appropriate sections before making your selection.

☒ Life sciences ☐ Behavioural & social sciences ☐ Ecological, evolutionary & environmental sciences

For a reference copy of the document with all sections, see nature.com/documents/nr-reporting-summary-flat.pdf

Life sciences study design

All studies must disclose on these points even when the disclosure is negative.

Sample size	n/a
Data exclusions	n/a
Replication	mES cell lines stably expressing a human PALB2 variant were tested for HR efficiency (DR-GFP), PARPi and cisplatin sensitivity, and G2/M checkpoint control at least twice. Variants showing variation in the outcome of either assay were analyzed in a third replicate experiment<br><br>In human cell lines, CRISPR-LMNA (U2OS), PARPi (HeLa) and RAD51 foci (HeLa) experiments were performed in triplicate.
Randomization	Variants were categorized as truncating based on the nature of the mutation, i.e. frameshift and nonsense variants. Likely benign missense variants were categorized as likely benign based on their frequency in the general population, i.e. between 0.1-15% based on the 1000 Genomes Project.
Blinding	n/a

Reporting for specific materials, systems and methods

We require information from authors about some types of materials, experimental systems and methods used in many studies. Here, indicate whether each material, system or method listed is relevant to your study. If you are not sure if a list item applies to your research, read the appropriate section before selecting a response.

Materials & experimental systems

n/a	Involved in the study	
☐	☒	Antibodies
☐	☒	Eukaryotic cell lines
☒	☐	Palaeontology
☒	☐	Animals and other organisms
☒	☐	Human research participants
☒	☐	Clinical data

Methods

n/a	Involved in the study	
☒	☐	ChIP-seq
☐	☒	Flow cytometry
☒	☐	MRI-based neuroimaging

Antibodies

Antibodies used

1. Rabbit polyclonal antibody against N-terminus of human PALB2 for detection in mES cells (test aliquot provided by Cell Signaling Technology prior to commercialization)
2. Homemade rabbit antibody against N-terminus of mouse PALB2 (NB3, gift form Bing Xia)
3. Mouse monoclonal antibody against alpha tubulin for detection in mouse cells (Sigma, T6199 clone DM1A)
4. Mouse monoclonal anti-GFP (Roche, #11814460001)
5. Anti-alpha tubulin for detection in human cells (Abcam, #ab7291)
6. Homemade rabbit polyclonal antibody against human PALB2 for detection in human cells (Jean-Yves Masson)
7. Homemade rabbit antibody against human BRCA1 (gift form Dan Durocher)
8. Rabbit anti-phospho-H3 Ser10 antibody (Sigma-Aldrich, 06-570)
9. Alexa-488 goat anti-rabbit antibody (Thermo Fischer, 11034)

Validation

1. Anti-human PALB2 from Cell Signaling was validated by us in this study. Western blot analysis only detected full-length human PALB2 protein in PALB2 knockout mES cells stably expressing human PALB2 cDNA, while this was not seen in cells expressing an empty vector control
2. Anti-mouse PALB2 from Bing Xia was validated by us and in a previous study (Simhadri et. al., J Biol. Chem., 2014,). Our Western blot analysis showed expression of endogenous PALB2 in wildtype mES cells, which was undectable in PALB2 knockout mES cells
3. Anti-alpha tubulin (Sigma, T6199 clone DM1A) was validated by supplier
4. Anti-GFP (Roche) was validated by supplier
5. Anti-alpha tubulin (Abcam, #ab7291) was validated by supplier
6. Homemade anti-human PALB2 was validated in a previous study (Xia et. al., Mol Cell, 2006).

7. Anti-human BRCA1 (Dan Durocher) was validated in a previous study (Noordermeer et. al., Nature, 2018)
8. Rabbit anti-phospho-H3 Ser10 antibody was validated by supplier
9. Alexa-488 goat anti-rabbit antibody was validated by supplier

Eukaryotic cell lines

Policy information about cell lines

Cell line source(s)	1. IB10 mouse ES cells, subclone from E14 (Robanus-Maandag et. al., Genes & Dev., 1998.)<br>2. U2OS and HeLa cells were purchased from ATCC
Authentication	Human U2OS and HeLa cells were authenticated using Short Tandem Repeat (STR) analysis by ATCC services (100% match)
Mycoplasma contamination	All cell lines were routinely and regularly tested for mycoplasma
Commonly misidentified lines<br>(See ICLAC register)	n/a

Flow Cytometry

Plots

Confirm that:

☐ The axis labels state the marker and fluorochrome used (e.g. CD4-FITC).

☐ The axis scales are clearly visible. Include numbers along axes only for bottom left plot of group (a 'group' is an analysis of identical markers).

☐ All plots are contour plots with outliers or pseudocolor plots.

☒ A numerical value for number of cells or percentage (with statistics) is provided.

Methodology

Sample preparation	2 days after I-Sce-I transfection or PARPi treatment mES cells were taken up PBS supplemented with 2% FBS
Instrument	ACEA NovoCyte flow cytometer
Software	NovoExpress 1.2.5
Cell population abundance	n/a
Gating strategy	To sort GFP and mCherry positive mES cells in DR-GFP experiments, three initial gates were set in the following sequential plots: 1) SSC-H scatter (Y-axis) set out against FSC-H scatter (X-axis), allowing us to set gate P1, 2) FSC-H scatter (Y-axis) set out against FSC-A scatter (X-axis), allowing us to set gate P2, and 3) SSC-H scatter (Y-axis) set out against SSC-A scatter (X-axis), allowing us to set gate P3. In a fourth plot (mCherry-H on Y-axis and FSC-H on X-axis), mCherry positive cells were scored using a gating based on mCherry negative control cells. In a fifth plot (GFP-H on Y-axis and FSC-H on X-axis), GFP positive cells were scored using a gating based on GFP negative control cells.<br><br>For PARPi and cisplatin sensitivity assays in mES cells, we made use of the NovoCyte flow cytometer to gate and count the living mES cells after PARPi treatment. Two sequential plots were generated: 1) SSC-H scatter (Y-axis) set out against FSC-H scatter (X-axis), allowing us to set gate P1, and 2) SSC-A scatter (Y-axis) set out against FSC-A scatter (X-axis), allowing us to set gate P2. Cell numbers for gate P2 were used to calculate percentages of viable cells after PARPi treatment.<br><br>To sort mitotic phoshpo H3 Ser10-positive mES cells in G2/M checkpoint experiments, three initial gates were set in the following sequential plots: 1) SSC-H scatter (Y-axis) set out against FSC-H scatter (X-axis), allowing us to set gate P1, 2) FSC-H scatter (Y-axis) set out against FSC-A scatter (X-axis), allowing us to set gate P2, and 3) SSC-H scatter (Y-axis) set out against SSC-A scatter (X-axis), allowing us to set gate P3. In a fourth plot (GFP-A on Y-axis and PI-A on X-axis), mitotic cells were scored.

☐ Tick this box to confirm that a figure exemplifying the gating strategy is provided in the Supplementary Information.
